# Supplementary material for: Towards Mapping of the Human Brain N-Glycome with Standardized Graphitic Carbon Chromatography
Source: Biomolecules. 2022 Jan 6;12(1):85. doi: 10.3390/biom12010085 (PMC8774104; doi:10.3390/biom12010085)
Supplement: Supplementary file 1 [file biomolecules-12-00085-s001.zip › biomolecules-1532158-SI.pdf]

**Supplementary Methods**

**N-Glycan Preparation from Human Brain Tissue**

About 500 mg brain tissue was cut into tiny pieces with the help of a scalpel. The pieces were transferred into a Falcon tube and mixed with 100 mM ammonium bicarbonate buffer containing 15 mM dithiothreitol (DTT) and 2.5 % sodium dodecyl sulfate in a total volume of 3.5 mL. After homogenization with an Ultra-Turrax T25 disperser the homogenate was boiled at 95°C for 5 min in order to eliminate enzymatic activities. After centrifugal clarification the supernatant was mixed with four volumes of methanol, one volume of chloroform and three volumes of water in the given order. After centrifugation of the mixture for 3 min at 2500 rpm, the upper phase was removed and another four volumes of methanol were added. After another centrifugation, the pellet was washed with methanol. The dried pellet was taken up in 50 mM ammonium acetate of pH 8.4 containing 1.25 M urea. For N-glycan release 2.5 U of PNGase F was added and the resulting mixture was incubated overnight at 37°C. This reaction mixture was loaded onto a Hypersep C18 cartridge (25 mg; Thermo Scientific, Vienna) that had been previously washed with 500 µL of methanol, then 500 µL 50% acetonitrile in 65 mM ammonium formate buffer (pH 3.0) and equilibrated twice with 500 µL 0.1% formic acid. The flow-through of the applied sample was collected and subjected to centrifugal evaporation. Reduction of the glycans was carried out in 1% sodium borohydride at room temperature overnight. Desalting was performed using HyperSep Hypercarb solid-phase extraction cartridges (25 mg) (Thermo Scientific, Vienna).

**N-Glycan Preparation from Pig Brain**

N-glycans from pig brain and white beans (ca. 50 g) were prepared by pepsin digestion, (glyco)-peptide extraction on a cation exchange resin and PNGase A treatment as previously described [1]. Peptic glycopeptides from bovine fibrin were desialylated in 50 mM sulfuric acid for 1 h at 80 °C. After neutralization with NaOH and centrifugation the glycopeptides were enriched by gel filtration and deglycosylated [1]. Reduction and final purification were done as described above.

**N-glycan Preparation from Purified Human IgG**

One hundred mg glycoprotein were dissolved in 10 mL 50 mM ammonium acetate (pH 8.4) and brought to 95 °C for 10 min. After cooling, 0.2 mU peptide:N-glycosidase F was added and digestion was carried out overnight at 37 °C. Subsequently, the released N-glycans were acidified, reduced and recovered by passage over Hypersep C18 cartridge as described above for small scale brain glycan preparations. IgG N-glycans were in part fractionated by semi-preparative HPLC on a Hypercarb column as described previously [1]. The major components $GnGnF^6$, $A^4GnF^6$, $GnA^4F^6$ and $A^4A^4F^6$ eluted in this order [2] and could be obtained as isolated fractions for the preparation of defined isomers.

**Preparation of $^{13}C_6$-UDP-Galactose**

UDP-$^{13}C_6$-galactose was prepared by incubation of $^{13}C_6$-galactose with galactokinase (Sigma-Aldrich, Vienna, Austria). Galactose-1-phosphate was converted to the nucleotide sugar in the presence of UDP-glucose by human galactose-1-phosphate uridylyltransferase, which was recombinantly expressed in Escherichia coli BL21 and purified via its His6-tag. The UDP-$^{13}C_6$-galactose was finally purified by PGC chromatography with a slightly alkaline buffer as described [3].

**Biosynthesis of Standard Glycan Structures**

The reduced and dried glycans, prepared as described above, were used as scaffolds for the different glycosidases and glycosyltransferases. The glycan standards were biosynthesized as described in Figures S1-S10. Fucosyltransferases were used with 1 mM GDP-fucose and 10 mM $MnCl_2$ in a total volume of 50 µL in 25 mM Tris/HCl pH 7.4 + 100 mM NaCl. Bovine β-1,4-galactosyltransferase, B4GalT1-Y285L and human β-1,3-galactosyltransferase were used with 1 mM UDP-galactose, $^{13}C_6$-UDP-galactose or 1 mM UDP-GalNAc and 20 mM $MnCl_2$ for bovine β-1,4-galactosyltransferase and B4GalT1-Y285L and 2 mM $MnCl_2$ for human β-1,3-galactosyltransferase in a total volume of 50 µL in 25 mM Tris/HCl pH 7.4 + 100 mM NaCl. N-acetylglucosaminyltransferases 1 and 3 were used with 1 mM UDP-GlcNAc and 20 mM $MnCl_2$ in 50 mM Tris/HCl pH 7.4. β-galactosidase from Aspergillus oryzae and fucosidase from bovine kidney were used in 0.1 M phosphate citrate buffer pH 5.0, $\alpha$- mannosidase from jack bean was used in sodium acetate buffer

pH 4.5 supplemented with 5 mM $ZnCl_2$. $\alpha$1,3/4-specific fucosidase, β1,3-specific galactosidase and $\alpha$1,6-mannosidase were used according to the manufacturer´s protocol.

All enzyme reactions were carried out overnight at 37 °C. After each enzyme-reaction step, the reaction mixtures were purified using carbon solid phase cartridges (Multi-Sep Hypercarb 25 mg, Thermo Scientific, Vienna) and the completeness of the enzymatic reaction was checked using PGC-LC-ESI-MS before further enzymatic processing. After the last enzymatic processing step, the finished glycan standards were again purified using PGC-SPEs, dried by centrifugal evaporation, taken up in a small volume of HQ-water and stored at -20°C until measurement.

**Mass Spectrometric Methods**

The purified samples were loaded on a PGC-column (100 mm × 0.32 mm, 5 µm particle size, Thermo Scientific, Waltham, MA, USA) with 10 mM ammonium bicarbonate as the aqueous solvent A and 80 % acetonitrile in solvent A as solvent B. The gradient was as followed: 0 – 4.5 min 1% B, from 4.5 – 5.5 min 8% B, from 5.5 – 58 min 8 - 22% B, from 58 – 62.5 min 22 - 30% B, from 62.5 – 63.5 min 68% B, followed by an equilibration period at 1% B from 64 – 70 min. The flowrate was 6 µL/min. Detection was performed with an ion trap mass spectrometer equipped with the standard ESI source directly linked to the Thermo Ultimate 3000 UPLC system. MS-scans were recorded from 400 – 1600 *m/z*. All samples were measured with an ion trap instrument (amaZon speed ETD; Bruker, Bremen, Germany) in positive mode or, in the case for some brain glycan runs, in negative mode. The negative mode measurements were carried out with the ICC target set to 100000, maximum accumulation time set to 250 ms and a scan range from 600 – 1500 *m/z*. The scan mode was set to enhanced resolution and the SPS (Smart Parameter Settings) were set to target mass 950 *m/z*, compound stability 100% and trap drive level 100%. For MS/MS, the number of precursor ions was set to 3 with active exclusion (exclusion after 1 spectrum and release after 0.22 min). The absolute treshhold was set to 10000 and the inclusion list was set from 700 – 1200 m/z. The scan mode for MS/MS was set to enhanced resolution with the ICC target set to 200000 and maximum accumulation time of 100 ms with isolation width set to 2 *m/z*. Smart frag was enabled in enhanced mode (start amplitude: 150%, end amplitude 200% and fragmentation time 35 ms). PAN was enabled with Low CID CutOff at 17% and time stretch at 250%. FxD was enabled with activation at 100%, decay at 100% and tickle level at 100%.

The positive mode measurements were carried out with the ICC target set to 165000, the maximum accumulation time set to 200 ms and a scan range from 600 – 1500 *m/z*. The scan mode was set to enhanced resolution and the SPS (Smart Parameter Settings) were set to target mass 850 *m/z*, compound stability 100 % and trap drive level 100%. For MS/MS, the number of precursor ions was set to 3 with active exclusion (exclusion after 1 spectrum and release after 0.25 ms). The absolute threshold was set to 20000 and the inclusion list to 700 – 1200 *m/z*. The scan mode for MS/MS was set to enhanced resolution with the ICC target set to 200000, the maximum accumulation time to 55 ms and the isolation width to 3 *m/z*. Smart Frag was enabled in enhanced mode (start amplitude 80%, end amplitude 120% and fragmentation time 20 ms). PAN and FxD was disabled for positive mode MS/MS. Standard source settings (capillary voltage 4.5 kV, nebulizer gas pressure 0.5 bar, drying gas 5 L/min, 200 °C) were used. Instrument tuning was optimized for a low mass range (around 1500-2000 Da molecules). MS/MS was carried out in data dependent acquisition mode (switching to MS/MS mode for eluted peaks). Data interpretation was done with DataAnalysis 4.0 (Bruker, Bremen, Germany).

## Supplementary Tables

**Table S1: Overview over all relevant observed glycan peaks of the neutral and desialylated charged fractions of the human brain.** The composition-vimin of 220-16.5 means that this structure is composed of two hexose-residues, two *N*-acetylhexosamine-residues and zero fucose-residues and elutes at 16.5 vimin. Available relevant MS/MS diagnostic fragments and other relevant features are shown in the column "CID/features" [4]. NA written after the composition means that these glycans eluted after the elution range covered by our internal standards and that therefore the normalized retention time could not be determined.

| Neutral fraction | | | | | |
|---|---|---|---|---|---|
| Composition-vimin | Calculated mass | Experimental mass | Abbreviation | Cartoon | CID/Features |
| 220-16.5 | 751.30 (H+) | 751.5 (H+) | MU | 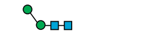 | 341 |
| 221-28.1 | 897.36 (H+) | 897.4 (H+) | MUF$^{6}$ | 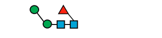 | 341, 350 |
| 320-26.5 | 913.35 (H+) | 913.4 (H+) | MM | 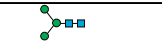 | |
| 321-36.5 | 1059.41 (H+) | 1059.4 (H+) | MMF$^{6}$ | 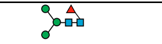 | 323, 350 |
| 431-31.7 | 712.77 (2H+) | 786.0 (2H+) | (AF)M | 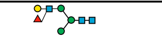 | |
| 431-NA (late eluting) | 712.77 (2H+) | 786.0 (2H+) | AMF$^{6}$ | 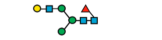 | 688, 670, 350 |
| 432-41.8 | 785.80 (2H+) | 786.0 (2H+) | (AF)MF$^{6}$ | 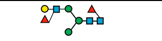 | 834, 350 |
| 432-42.5 | 785.80 (2H+) | 786.0 (2H+) | M(AF)F$^{6}$ | 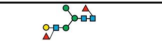 | |
| 341-14.7 | 733.29 (2H+) | 733.4 (2H+) | GnMF$^{6}$bi | 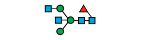 | 508, 350 |
| 341-28.1 | 733.29 (2H+) | 733.5 (2H+) | GnGnF$^{6}$ | 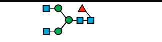 | 508, 350 |
| 341-29.2 | 733.29 (2H+) | 733.4 (2H+) | ? | ? | 526, 508, 350 |
| 341-37.2 | 733.29 (2H+) | 733.5 (2H+) | ? | ? | 526, 350 |
| 350-11.5 | 761.80 (2H+) | 762.0 (2H+) | GnGnbi | 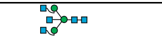 | |
| 351-17.2 | 834.83 (2H+) | 834.9 (2H+) | GnGnF$^{6}$bi | 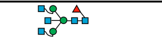 | |
| 351-27.2 | 834.83 (2H+) | 835.0 (2H+) | AnMF$^{6}$bi | 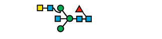 | 407 (pos), 729, 711, 350 |
| 352-29.4 | 907.86 (2H+) | 907.9 (2H+) | (AnF)GnF$^{6}$/(AnF)MF$^{6}$bi | 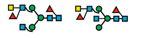 | 407 (pos), 553 (pos) |
| 361-24.1 | 936.37 (2H+) | 936.4 (2H+) | GnAnF$^{6}$bi/AnGnF$^{6}$bi | 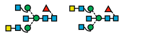 | 407 (pos) |
| 361-35.3 | 936.37 (2H+) | 936.4 (2H+) | AnAnF$^{6}$ | 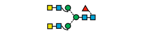 | 407 (pos) |
| 362-17.7 | 1009.40 (2H+) | 1009.4 (2H+) | Gn(AnF)F$^{6}$bi/(AnF)GnF$^{6}$bi | 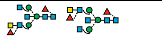 | 839, 857, 490, 508 |
| 362-32.9 | 1009.40 (2H+) | 1009.4 (2H+) | An(AnF)F$^{6}$/(AnF)AnF$^{6}$ | 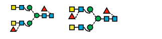 | 857, 875 |
| 440-11.2 | 741.28 (2H+) | 741.4 (2H+) | M$^{3}$Gnbi | 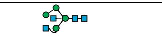 | Fucosidase digestion |
| 441-15.5 | 814.31 (2H+) | 814.5 (2H+) | M$^{3}$GnF$^{6}$bi | 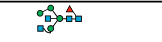 | 467, 350 |
| 441-20.5 | 814.31 (2H+) | 814.4 (2H+) | (AF)Gn/Gn(AF) | 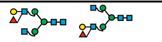 | |
| 441-32.6 | 814.31 (2H+) | 814.5 (2H+) | A$^{4}$GnF$^{6}$ | 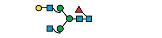 | |
| 441-33.8 | 814.31 (2H+) | 814.5 (2H+) | GnA$^{4}$F$^{6}$ | 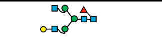 | |
| 442-25.0 | 887.34 (2H+) | 887.4 (2H+) | M$^{3}$(AnF)F$^{6}$ | 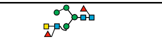 | |
| 442-29.6 | 887.34 (2H+) | 887.5 (2H+) | Gn(AF)F$^{6}$/(AF)GnF$^{6}$ | 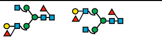 | |
| 442-37.8 | 887.34 (2H+) | 887.5 (2H+) | ? | ? | |
| 451-12.0 | 915.85 (2H+) | 915.9 (2H+) | (AF)Gnbi/Gn(AF)bi | 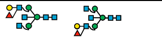 | |
| 451-18.8 | 915.85 (2H+) | 916.0 (2H+) | A$^{4}$GnF$^{6}$bi | 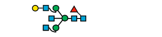 | |
| 451-19.8 | 915.85 (2H+) | 916.0 (2H+) | GnA$^{4}$F$^{6}$bi | 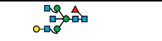 | |
| 451-27.4 | 915.85 (2H+) | 915.9 (2H+) | ? | ? | |

| Desialylated fraction | | | | | |
|---|---|---|---|---|---|
| 351-32.5 | 834.83 (2H+) | 834.9 (2H+) | AnGnF$^{6}$ | 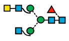 | 407 (pos), 350, 465 |
| 440-25.2 | 741.28 (2H+) | 741.4 (2H+) | ? | ? | |
| 441-27.2 | 814.31 (2H+) | 814.4 (2H+) | M$^{3}$AnF6 | 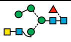 | 407 (pos), 467, 350, 465 |
| 441-32.9 | 814.31 (2H+) | 814.5 (2H+) | A$^{4}$GnF$^{6}$ | 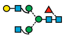 | |
| 441-33.7 | 814.31 (2H+) | 814.4 (2H+) | GnA$^{4}$F$^{6}$ | 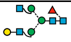 | |
| 441-35.1 | 814.31 (2H+) | 814.5 (2H+) | A$^{3}$GnF$^{6}$ | 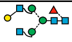 | b1,3-galactosidase |
| 451-18.9 | 915.85 (2H+) | 916.0 (2H+) | A$^{4}$GnF$^{6}$bi | 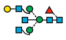 | |
| 451-20.1 | 915.85 (2H+) | 916.0 (2H+) | GnA$^{4}$F$^{6}$bi | 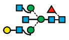 | |
| 451-23.3 | 915.85 (2H+) | 915.9 (2H+) | (AF)An | 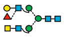 | 407 (pos) |
| 451-31.8 | 915.85 (2H+) | 915.9 (2H+) | ? | ? | |
| 451-33.9 | 915.85 (2H+) | 915.9 (2H+) | ? | ? | 407 (pos) |
| 451-34.8 | 915.85 (2H+) | 915.9 (2H+) | AnA$^{4}$F$^{6}$ | 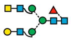 | 711, 350, 424 |
| 452-32.9 | 988.88 (2H+) | 989.0 (2H+) | (AF)AnF$^{6}$ | 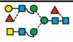 | 816, 350, 407 (pos) |
| 540-22.7 | 822.31 (2H+) | 822.4 (2H+) | ? | ? | |
| 540-27.9 | 822.31 (2H+) | 822.4 (2H+) | A$^{4}$A$^{4}$ | 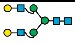 | |
| 541-22.7 | 895.34 (2H+) | 895.5 (2H+) | M$^{3}$GnF$^{6}$-Abi | 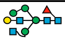 | |
| 541-25.4 | 895.34 (2H+) | 895.4 (2H+) | (AF)A$^{4}$ | 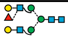 | |
| 541-36.2 | 895.34 (2H+) | 895.5 (2H+) | A$^{4}$A$^{4}$F$^{6}$ | 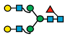 | |
| 542-32.7 | 968.37 (2H+) | 968.4 (2H+) | (AF)A$^{4}$F$^{6}$ | 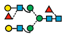 | |
| 542-34.0 | 968.37 (2H+) | 968.5 (2H+) | A$^{4}$(AF)F$^{6}$ | 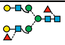 | |
| 542-35.5 | 968.37 (2H+) | 968.5 (2H+) | (AF)A$^{3}$F$^{6}$/A$^{3}$(AF)F$^{6}$ | 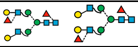 | b1,3-galactosidase |
| 550-15.1 | 923.85 (2H+) | 923.9 (2H+) | A$^{4}$A$^{4}$bi | 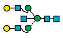 | |
| 551-22.3 | 996.88 (2H+) | 997.0 (2H+) | A$^{4}$A$^{4}$F$^{6}$bi | 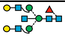 | |
| 551-33.3 | 996.88 (2H+) | 996.9 (2H+) | ? | ? | Triantennary |
| 551-35.0 | 996.88 (2H+) | 996.9 (2H+) | ? | ? | Triantennary |
| 551-37.4 | 996.88 (2H+) | 996.9 (2H+) | ? | ? | Triantennary |
| 552-19.9 | 1069.91 (2H+) | 1069.9 (2H+) | A$^{4}$(AF)F$^{6}$bi/(AF)A$^{4}$F$^{6}$bi | 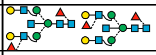 | |
| 552-30.4 | 1069.91 (2H+) | 1069.9 (2H+) | ? | ? | Triantennary |
| 552-32.5 | 1069.91 (2H+) | 1069.9 (2H+) | ? | ? | Triantennary |
| 552-37.0 | 1069.91 (2H+) | 1069.9 (2H+) | ? | ? | Triantennary |
| 362-17.7 | 1009.40 (2H+) | 1009.4 (2H+) | (AnF)GnF$^{6}$bi/Gn(AnF)F$^{6}$bi | 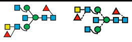 | 407 (pos), 553 (pos) + negative mode (Figure 3) |
| 362-32.4 | 1009.40 (2H+) | 1009.4 (2H+) | An(AnF)F$^{6}$/(AnF)AnF$^{6}$ | 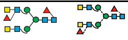 | 407 (pos), 553 (pos) + negative mode (Figure 3) |

**Table S2: Overview over all biosynthesized glycan standards.** A composition of 341 means that this structure is composed of three hexose-residues, four *N*-acetylhexose-residues and one fucose-residue.

| Composition | Abbreviation | Cartoon | vimin | Composition | Abbreviation | Cartoon | vimin |
|---|---|---|---|---|---|---|---|
| 341 | GnGnF$^{6}$ | | 28.1 | 541 | GnA$^{3\text{-}3}$F$^{6}$ | | 44.1 |
| 440 | GnA$^{4}$ | | 26.1 | 542 | M$^{3}$GnF$^{6}$(AF)-bi | | 15 |
| 440 | A$^{4}$Gn | | 27.2 | 542 | (AF)(AF) | | 20.9 |
| 441 | A$^{4}$GnF$^{6}$ | | 32.5 | 542 | (FA)(FA) | | 32.4 |
| 441 | GnA$^{4}$F$^{6}$ | | 33.7 | 542 | F$^{2\text{-}3}$F$^{2\text{-}3}$ | | 39.5 |
| 441 | A$^{3}$GnF$^{6}$ | | 35.1 | 542 | F$^{2\text{-}4}$F$^{2\text{-}4}$ | | 41 |
| 441 | GnA$^{3}$F$^{6}$ | | 35.8 | 542 | F$^{2\text{-}4}$F$^{2\text{-}3}$ | | 37.8 |
| 441 | (AF)Gn | | 20.2 | 542 | F$^{2\text{-}3}$F$^{2\text{-}4}$ | | 39.5 |
| 441 | Gn(AF) | | 20.2 | 542 | A$^{4}$(AF)F$^{6}$ | | 32.5 |
| 441 | Gn(FA) | | 24.7 | 542 | (AF)A$^{4}$F$^{6}$ | | 33.9 |
| 441 | (FA)Gn | | 28.2 | 542 | (AF)(FA) | | 23.9 |
| 441 | F$^{2\text{-}4}$Gn | | 31.2 | 542 | (FA)(AF) | | 28.7 |
| 441 | GnF$^{2\text{-}4}$ | | 31.2 | 542 | A$^{4}$(FA)F$^{6}$ | | 35.6 |
| 441 | F$^{2\text{-}3}$Gn | | 31 | 542 | (FA)A$^{4}$F$^{6}$ | | 40.6 |
| 441 | GnF$^{2\text{-}3}$ | | 32 | 542 | A$^{3}$(FA)F$^{6}$ | | 39.2 |
| 442 | (AF)GnF$^{6}$ | | 29.9 | 542 | (FA)A$^{3}$F$^{6}$ | | 41.9 |
| 442 | Gn(AF)F$^{6}$ | | 29.9 | 542 | (AF)A$^{3}$F$^{6}$ | | 35.4 |
| 442 | Gn(FA)F$^{6}$ | | 34 | 542 | A$^{3}$(AF)F$^{6}$ | | 35.4 |
| 442 | (FA)GnF$^{6}$ | | 37.2 | 543 | (AF)(AF)F$^{6}$ | | 30 |
| 540 | A$^{4}$A$^{4}$ | | 27.9 | 543 | (FA)(FA)F$^{6}$ | | 40.8 |
| 540 | A$^{4}$A$^{3}$ | | 29.3 | 543 | (AF)(FA)F$^{6}$ | - | - |
| 540 | A$^{3}$A$^{4}$ | | 31.8 | 543 | (FA)(AF)F$^{6}$ | - | - |
| 540 | A$^{3}$A$^{3}$ | | 32.5 | 350 | GnGnbi | | 11.5 |
| 540 | M$^{3}$A$^{4}$bi | | 11.9 | 350 | AnGn | - | - |
| 540 | Man5Gnbi | | 13 | 350 | GnAn | - | - |
| 540 | M$^{3}$A$^{3}$bi | | 13.7 | 351 | GnGnF$^{6}$bi | | 17 |
| 540 | M$^{3}$GnA-bi | | 15.7 | 351 | AnGnF$^{6}$ | | 32.2 |
| 540 | Man5An | | 32.4 | 351 | GnAnF$^{6}$ | | 31.8 |
| 540 | A$^{3\text{-}4}$Gn | | 30.4 | 360 | AnAn | | 28.1 |
| 540 | GnA$^{3\text{-}4}$ | | 33.4 | 360 | GnAnbi | - | - |
| 540 | A$^{3\text{-}3}$Gn | | 35.1 | 360 | AnGnbi | - | - |
| 540 | GnA$^{3\text{-}3}$ | | 35.9 | 361 | AnAnF$^{6}$ | | 35.7 |
| 541 | M$^{3}$Gn(AF)-bi | | 11.2 | 361 | GnAnF$^{6}$bi | | 19.3 |
| 541 | M$^{3}$A$^{4}$F$^{6}$bi | | 16.6 | 361 | AnGnF$^{6}$bi | | 19.3 |
| 541 | Man5GnF$^{6}$bi | | 17.5 | 372 | AnAnF$^{6}$bi | | 25.4 |

| | | | | | | | |
|---|---|---|---|---|---|---|---|
| 541 | M$^{3}$F$^{2-4}$bi | | 18.5 | 450 | GnA$^{4}$bi | | 19 |
| 541 | M$^{3}$A$^{3}$F$^{6}$bi | | 18.8 | 450 | A$^{4}$Gnbi | | 13.7 |
| 541 | A$^{4}$A$^{4}$F$^{3}$ | | 19.6 | 450 | GnA$^{3}$bi | | 15.3 |
| 541 | A$^{4}$A$^{3}$F$^{3}$ | | 19.8 | 450 | A$^{3}$Gnbi | | 13.1 |
| 541 | M$^{3}$GnF$^{2-4}$-bi | | 21.1 | 450 | AnA$^{4}$ | | 29.9 |
| 541 | M$^{3}$F$^{2-3}$bi | | 21.2 | 450 | A$^{4}$An | | 22.1 |
| 541 | Man5(AnF$^{3}$) | | 21.8 | 451 | GnA$^{4}$F$^{6}$bi | | 19 |
| 541 | M$^{3}$GnF$^{6}$A-bi | | 22.7 | 451 | A$^{4}$GnF$^{6}$ | | 20 |
| 541 | (A$^{3-4}$F$^{3}$)Gn | | 23.5 | 451 | GnA$^{3}$F$^{6}$bi | | 19.2 |
| 541 | A$^{4}$(AF) | | 23.9 | 451 | A$^{3}$GnF$^{6}$bi | | 22.2 |
| 541 | A$^{3}$A$^{4}$F$^{3}$ | | 24 | 451 | Gn(AF)bi | | 12 |
| 541 | A$^{3}$A$^{3}$F$^{3}$ | | 24 | 451 | (AF)Gnbi | | 12 |
| 541 | (AF)A$^{4}$ | | 25.4 | 451 | (FA)Gnbi | | 12.1 |
| 541 | Gn(A$^{3-4}$F$^{3}$) | | 26.2 | 451 | Gn(FA)bi | | 14.4 |
| 541 | (AF)A$^{3}$ | | 26.5 | 451 | AnA$^{4}$F$^{6}$ | | 34.9 |
| 541 | A$^{3}$(AF) | | 27.9 | 451 | A$^{4}$AnF$^{6}$ | | 36.9 |
| 541 | A$^{4}$(FA) | | 27.9 | 452 | Gn(AF)F$^{6}$bi | | 17 |
| 541 | A$^{3}$(FA) | | 31.2 | 452 | (AF)GnF$^{6}$bi | | 17.5 |
| 541 | F$^{2-4}$A$^{4}$ | | 31.6 | 452 | (FA)GnF$^{6}$bi | | 17.7 |
| 541 | Man5An$^{4}$F$^{6}$ | | 32.4 | 452 | Gn(FA)F$^{6}$bi | | 21.8 |
| 541 | (FA)A$^{4}$ | | 32.5 | 550 | A$^{4}$A$^{4}$bi | | 15.3 |
| 541 | F$^{2-4}$A$^{3}$ | | 32.9 | 550 | A$^{3}$A$^{3}$bi | | 18 |
| 541 | A$^{4}$F$^{2-4}$ | | 34.1 | 551 | A$^{4}$A$^{4}$F$^{6}$bi | | 22.3 |
| 541 | (FA)A$^{3}$ | | 34.5 | 551 | A$^{3}$A$^{3}$F$^{6}$bi | | 24.2 |
| 541 | F$^{2-3}$A$^{4}$ | | 35.6 | 551 | (AF)A$^{4}$bi | | 14 |
| 541 | A$^{4}$F$^{2-3}$ | | 36.2 | 551 | A$^{4}$(AF)bi | | 14 |
| 541 | A$^{4}$A$^{4}$F$^{6}$ | | 36.2 | 551 | (FA)A$^{3}$bi | | 15.5 |
| 541 | F$^{2-3}$A$^{3}$ | | 35.6 | 551 | A$^{3}$(FA)bi | | 15.5 |
| 541 | A$^{3}$F$^{2-3}$ | | 37.2 | 552 | (AF)(AF)bi | | 12.5 |
| 541 | A$^{4}$A$^{3}$F$^{6}$ | | 37.6 | 552 | (AF)A$^{4}$F$^{6}$bi | | 19.6 |
| 541 | A$^{3}$F$^{2-4}$ | | 37.8 | 552 | A$^{4}$(AF)F$^{6}$bi | | 19.6 |
| 541 | A$^{3-4}$GnF$^{6}$ | | 38.3 | 552 | A$^{3}$(FA)F$^{6}$bi | | 22 |
| 541 | A$^{3}$A$^{4}$F$^{6}$ | | 39.6 | 552 | (FA)A$^{3}$F$^{6}$bi | | 22 |
| 541 | A$^{3}$A$^{3}$F$^{6}$ | | 40.4 | 552 | (FA)(FA)bi | | - |
| 541 | GnA$^{3-4}$F$^{6}$ | | 41.1 | 553 | (AF)(AF)F$^{6}$bi | | 17.4 |
| 541 | A$^{3-3}$GnF$^{6}$ | | 42.5 | 553 | (FA)(FA)F$^{6}$bi | | 20.9 |

## Supplementary Figures

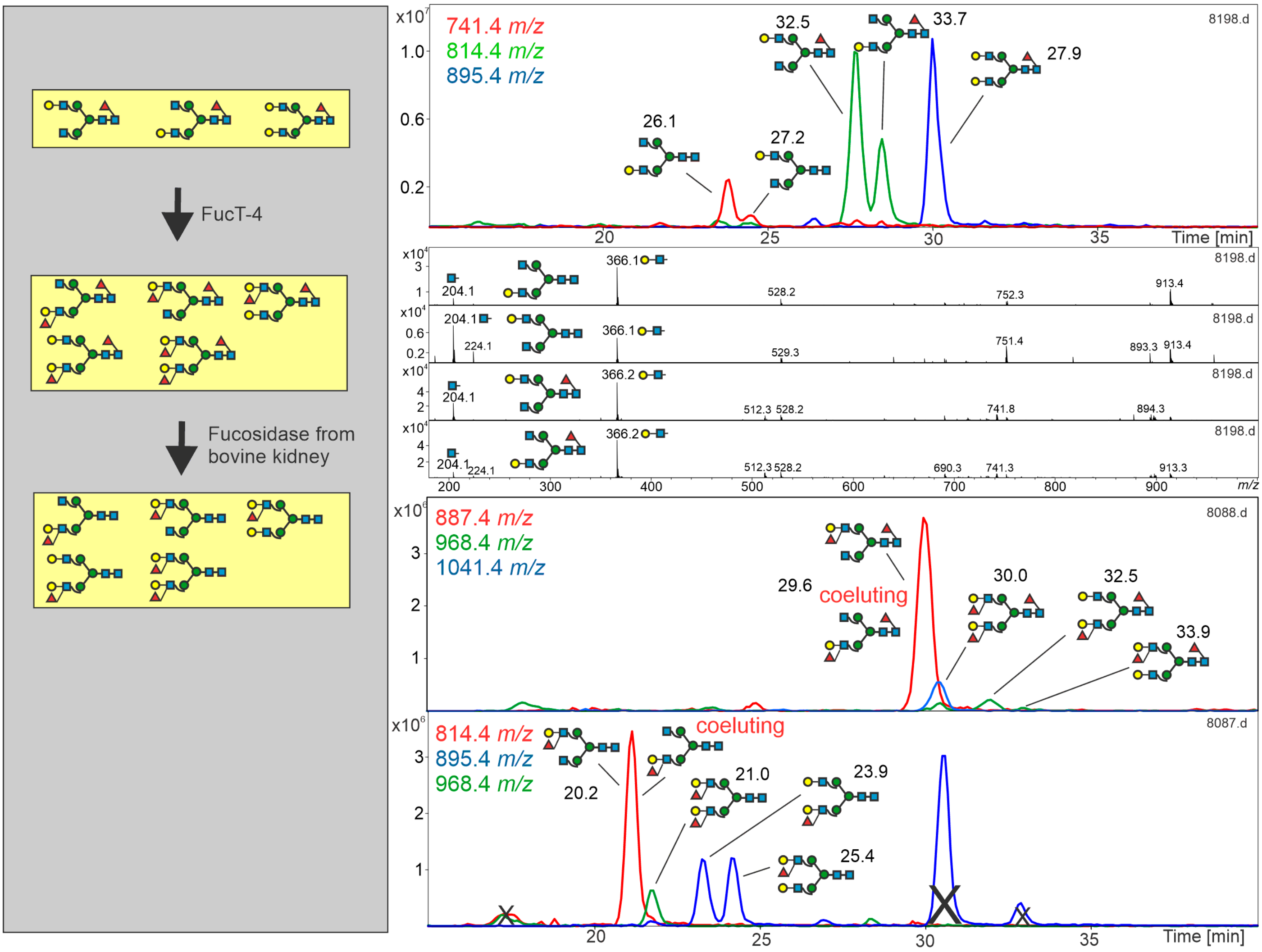


**Figure S1: Biosynthesis route of biantennary glycans with β1,4 linked galactose, core fucose and α1,3-linked fucose without bisecting GlcNAc.** PGC-ESI-MS chromatograms of the respective glycan structures and the corresponding positive mode MS/MS spectra are shown on the right side. Arm-isomers were assigned by posCID with the knowledge that the lower arm (α1,3-arm) detaches more easily than the upper arm (α1,6-arm). All peaks represent doubly charged ions of glycans.

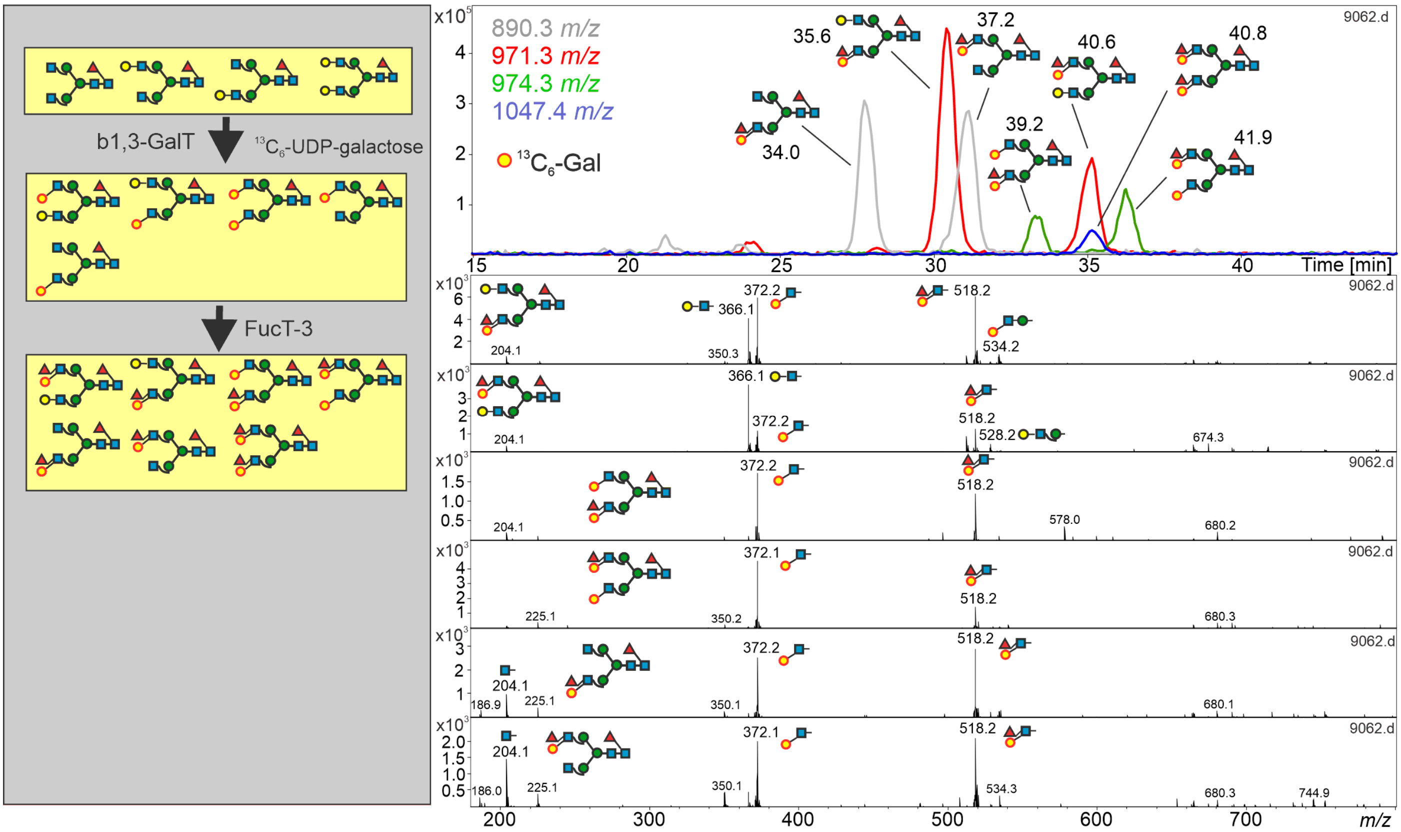


**Figure S2: Biosynthesis route of biantennary glycans with β1,4- and β1,3-linked galactose and α1,3 and α1,4-linked fucose without bisecting GlcNAc.** Galactose in β1,3-linkage to GlcNAc was labelled with $^{13}C_6$-galactose. PGC-ESI-MS chromatograms of the respective glycan structures and the corresponding positive mode MS/MS spectra are shown on the right side. Arm-isomers could be assigned with the knowledge that the lower arm (α1,3-arm) detaches more easily than the upper arm (α1,6-arm) and that the β1,3-linked galactose is isotope-labelled. All glycans occurred as doubly charged ions.

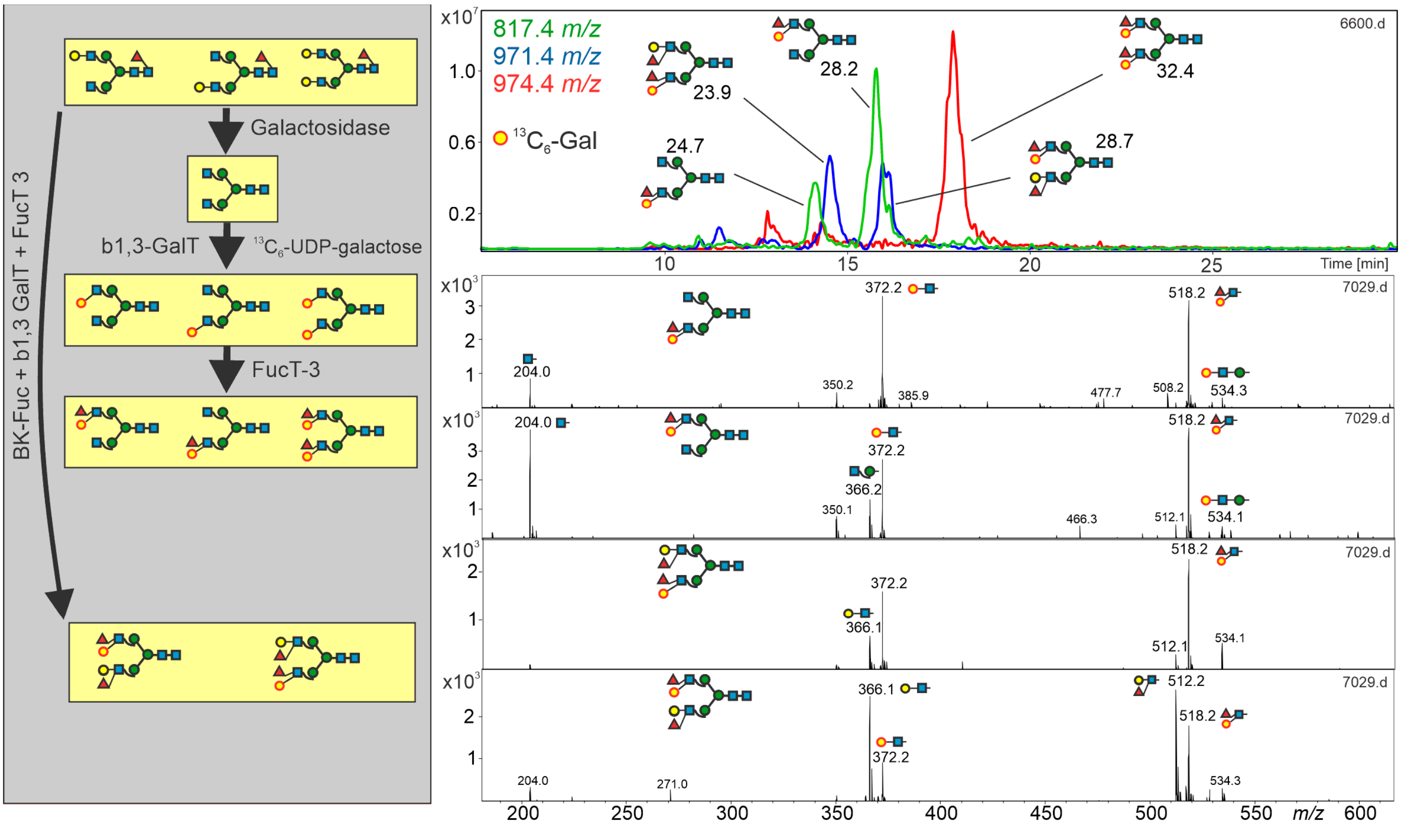

**Figure S3: Biosynthesis route of biantennary glycans with β1,4- and β1,3-linked galactose and core fucose and $\alpha$1,4-linked fucose without bisecting GlcNAc.** Galactose in β1,3-linkage to GlcNAc was labelled with $^{13}C_6$-galactose. PGC-ESI-MS chromatograms of the respective glycan structures and the corresponding positive mode MS/MS spectra are shown on the right side. Arm-isomers could be assigned with the knowledge that the lower arm ($\alpha$1,3-arm) detaches more easily than the upper arm ($\alpha$1,6-arm) and that the β1,3-linked galactose is isotope-labelled. All glycans shown are present as doubly charged ions.

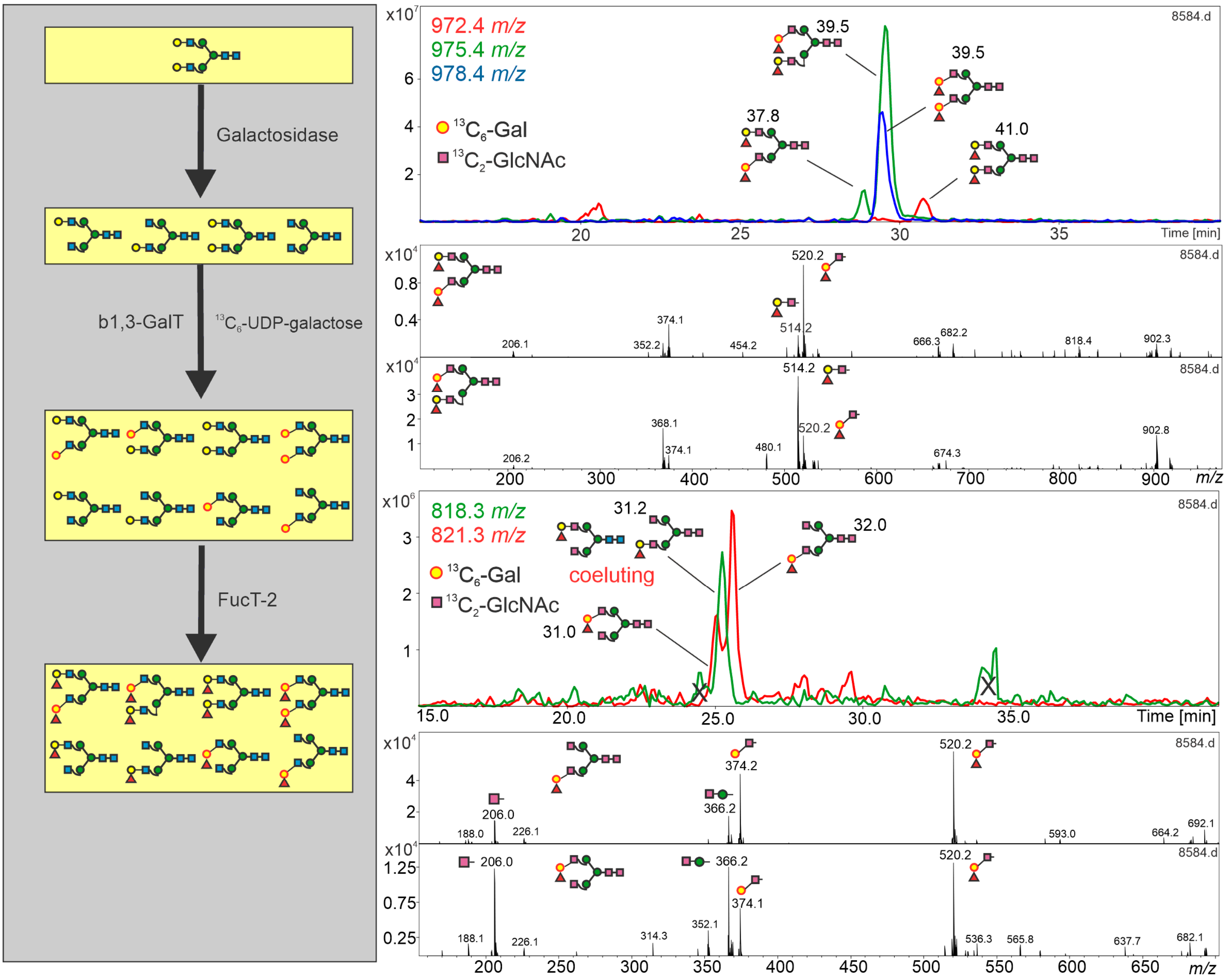


**Figure S4: Biosynthesis route of biantennary glycans with β1,4- and β1,3-linked galactose $\alpha$1,2-linked fucose to the galactose residue without bisecting GlcNAc.** Galactose linked to GlcNAc in β1,3-position is always labelled with 13C6-galactose. All GlcNAc residues are $^{13}C_2$-acetylated. PGC-ESI-MS chromatograms of the respective glycan structures and the corresponding positive mode MS/MS spectra are shown on the right side. Arm-isomers could be assigned with the knowledge that the lower arm ($\alpha$1,3-arm) detaches more easily than the upper arm ($\alpha$1,6-arm) and that the β1,3-linked galactose is isotope-labelled. All glycans shown are present as doubly charged ions.

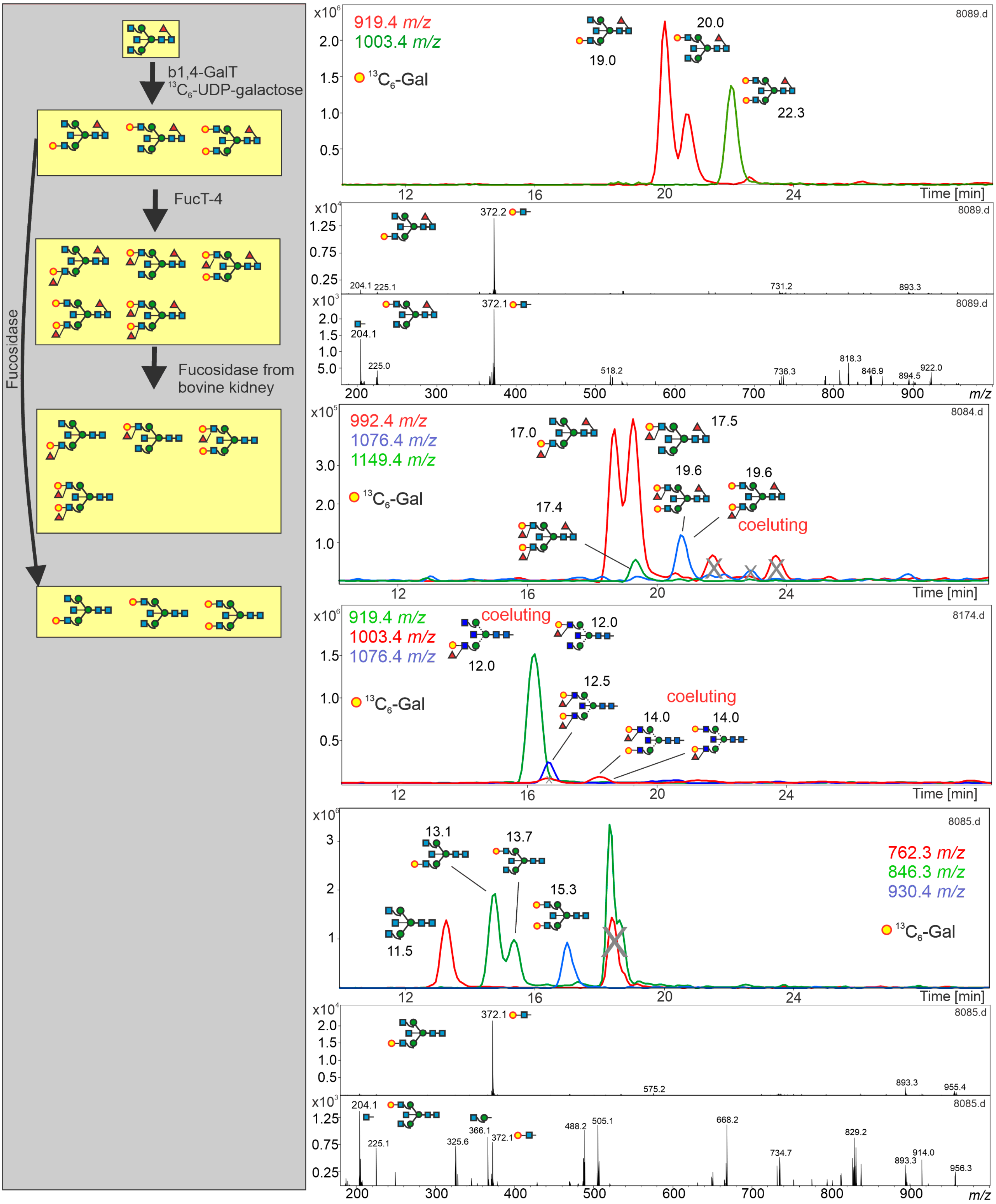


**Figure S5: Biosynthesis route of biantennary glycans with β1,4-galactose and core fucose and α1,3-linked fucose with bisecting GlcNAc.** GnGnF⁶bi was obtained by HILIC fractionation of porcine brain. All galactose residues are isotope-labelled ($^{13}C_6$-galactose). PGC-ESI-MS chromatograms of the respective glycan structures and the corresponding positive mode MS/MS spectra are shown on the right side. Arm-isomers could be assigned with the knowledge that the lower arm (α1,3-arm) detaches more easily than the upper arm (α1,6-arm). The glycans were reduced with NaBD4. All glycans shown are present as doubly charged ions.

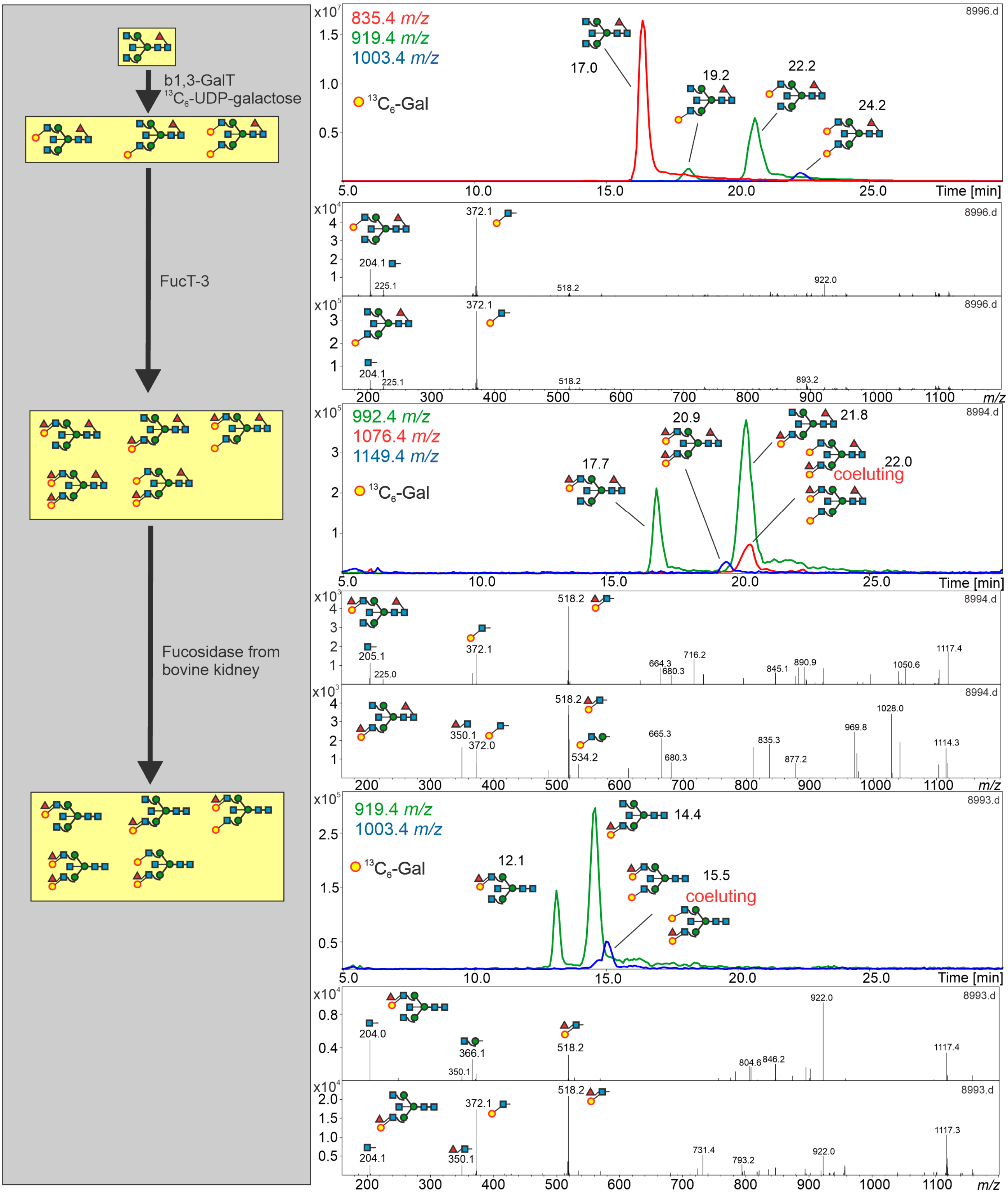


**Figure S6: Biosynthesis route of biantennary glycans with β1,3-galactose and core fucose and α1,4-linked fucose with bisecting GlcNAc.** $GnGnF^6bi$ was obtained by HILIC fractionation of porcine brain. All galactose residues are isotope-labelled ($^{13}C_6$-galactose). PGC-ESI-MS chromatograms of the respective glycan structures and the corresponding positive mode MS/MS spectra are shown on the right side. Arm-isomers could be assigned with the knowledge that the lower arm (α1,3-arm) detaches more easily than the upper arm (α1,6-arm) and that the β1,3-linked galactose is isotope-labelled. The glycans were reduced with NaBD4. All glycans shown are present as doubly charged ions.

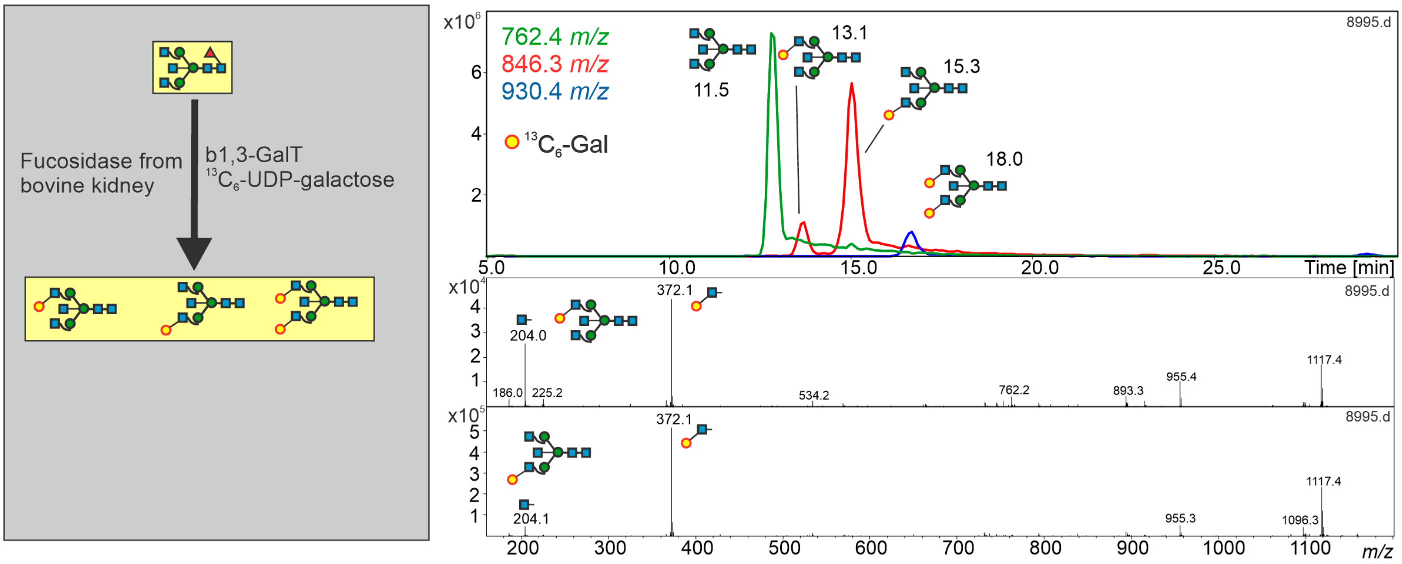


**Figure S7: Biosynthesis route of nonfucosylated biantennary glycans with β1,3-galactose and bisecting GlcNAc.** GnGnF[6]bi was obtained by HILIC fractionation of porcine brain- All galactose residues are isotope-labelled $^{13}C_6$-galactose). PGC-ESI-MS chromatograms of the respective glycan structures and the corresponding positive mode MS/MS spectra are shown on the right side. Arm-isomers could be assigned with the knowledge that the lower arm (α1,3-arm) detaches more easily than the upper arm (α1,6-arm) and that the β1,3-linked galactose is isotope-labelled. All glycans shown are present as doubly charged ions.

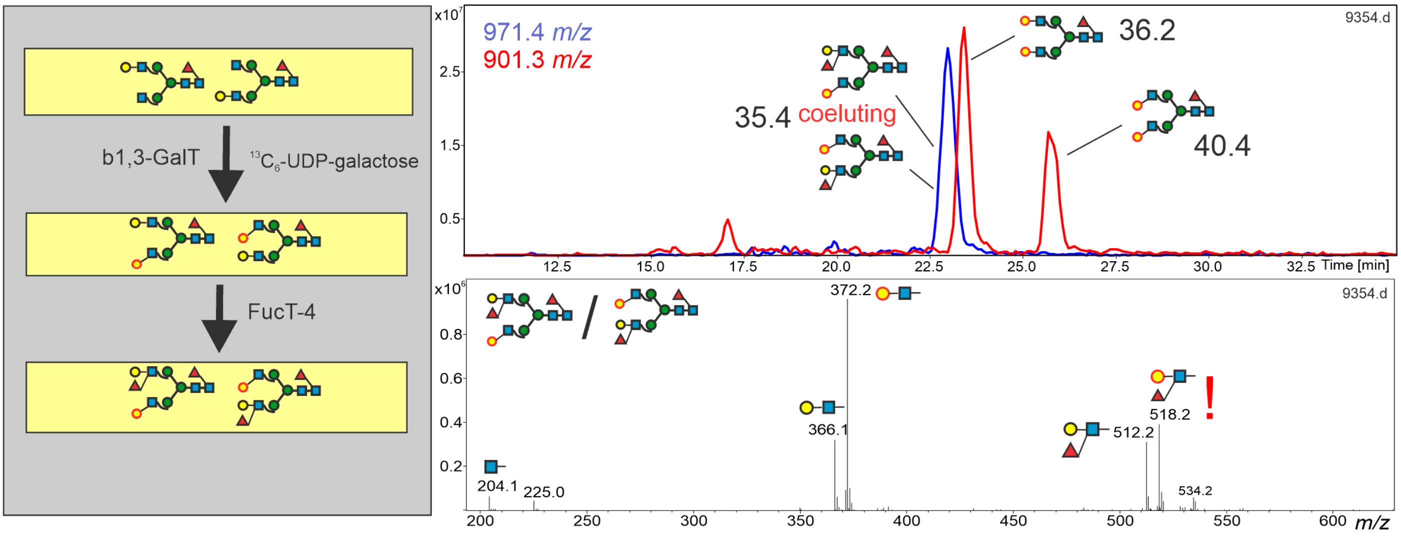


**Figure S8: Biosynthesis route of doubly fucosylated biantennary glycans with β1,3-galactose, β1,4-galactose, core fucose and α1,3-antenna fucose.** β1,3-galactose residues are isotope-labelled $^{13}C_6$-galactose. PGC-ESI-MS chromatograms of the respective glycan structures and the corresponding positive mode MS/MS spectra are shown on the right side. Arm-isomers could be assigned with the knowledge that the lower arm (α1,3-arm) detaches more easily than the upper arm (α1,6-arm) and that the β1,3-linked galactose is isotope-labelled. All glycans shown are present as doubly charged ions. The red exclamation mark denotes fragments derived from fucose migration.

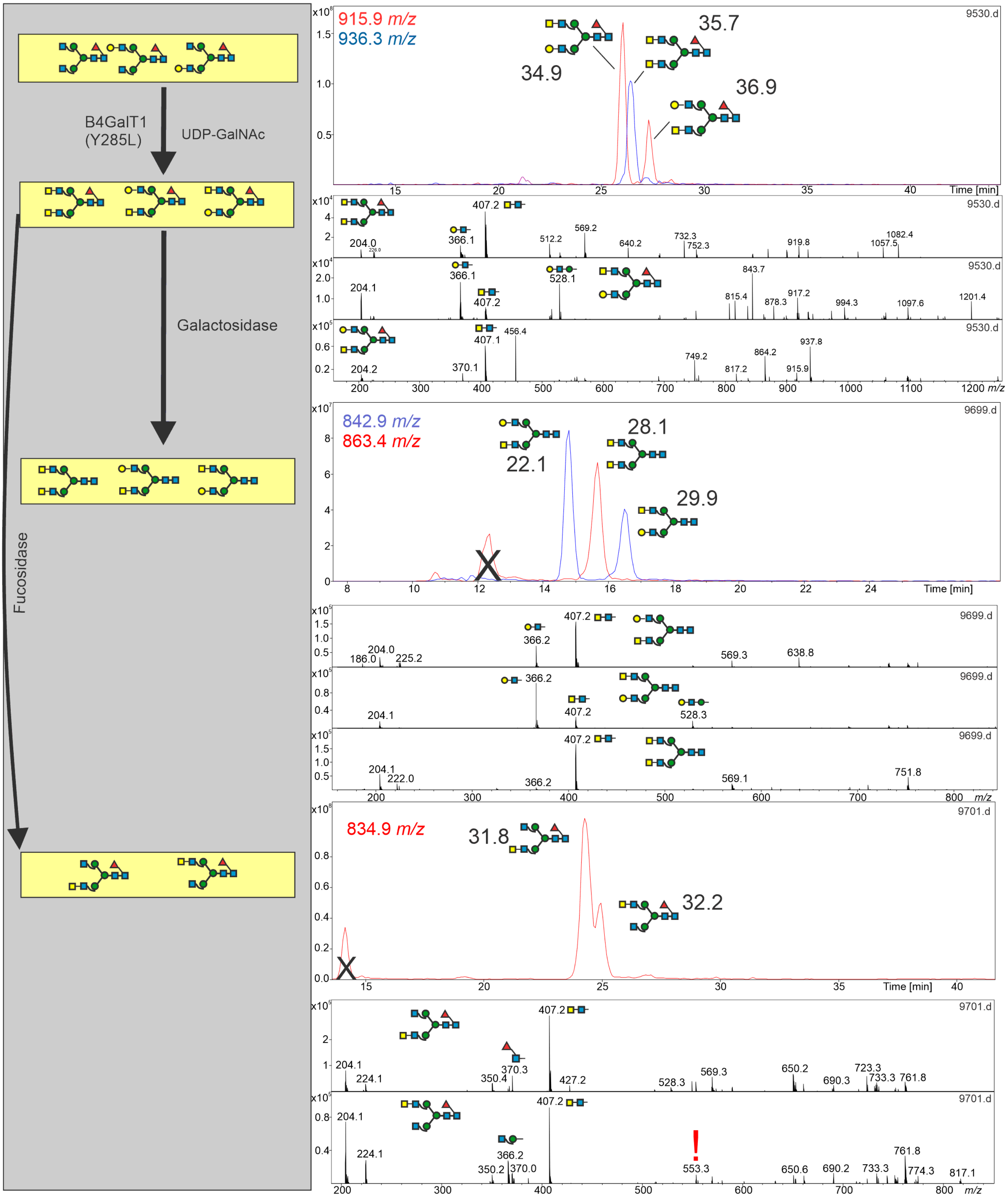


**Figure S9: Biosynthesis route of core-fucoylated biantennary glycans with LacdiNAc-epitopes**. PGC-ESI-MS chromatograms of the respective glycan structures and the corresponding positive mode MS/MS spectra are shown on the right side. Arm-isomers could be assigned with the knowledge that the lower arm (α1,3-arm) detaches more easily than the upper arm (α1,6-arm). All glycans shown are present as doubly charged ions. The red exclamation mark denotes fragments derived from fucose migration.

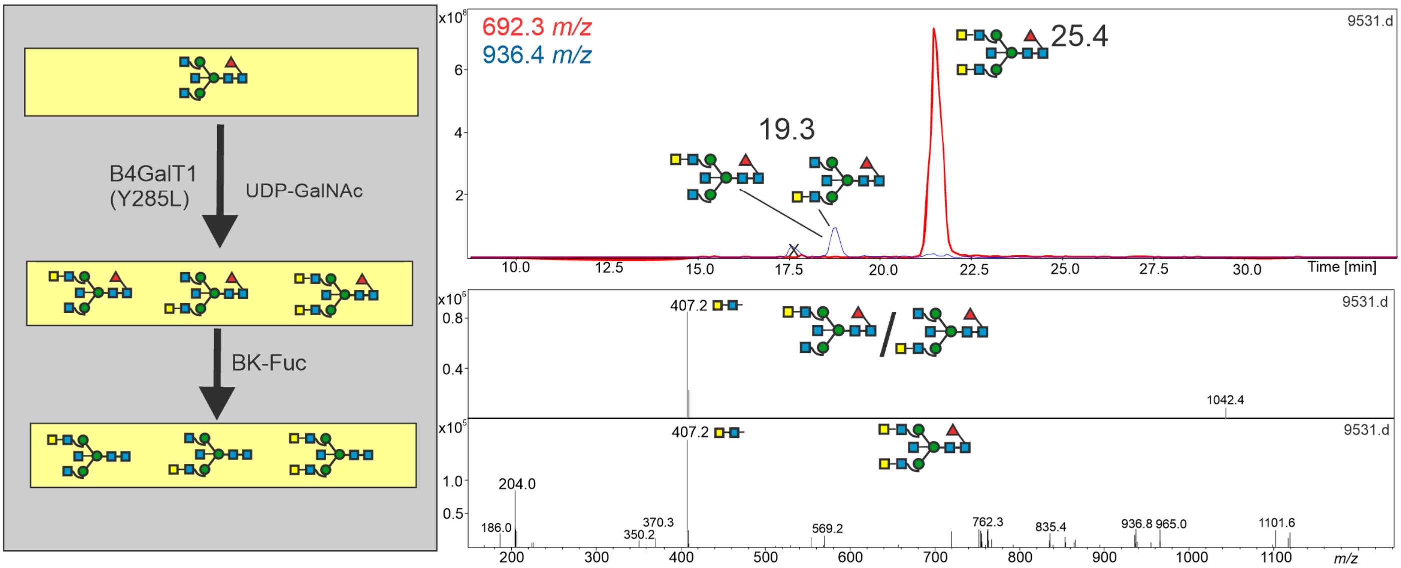


**Figure S10: Biosynthesis route of core-fucosylated biantennary glycans with LacdiNAc-epitopes and bisecting GlcNAc**. GnGnF⁶bi was obtained by HILIC fractionation of porcine brain. PGC-ESI-MS chromatograms of the respective glycan structures and the corresponding positive mode MS/MS spectra are shown on the right side. Arm-isomers could be assigned with the knowledge that the lower arm (α1,3-arm) detaches more easily than the upper arm (α1,6-arm). All glycans shown are present as doubly charged ions except for AnAnF⁶bi.

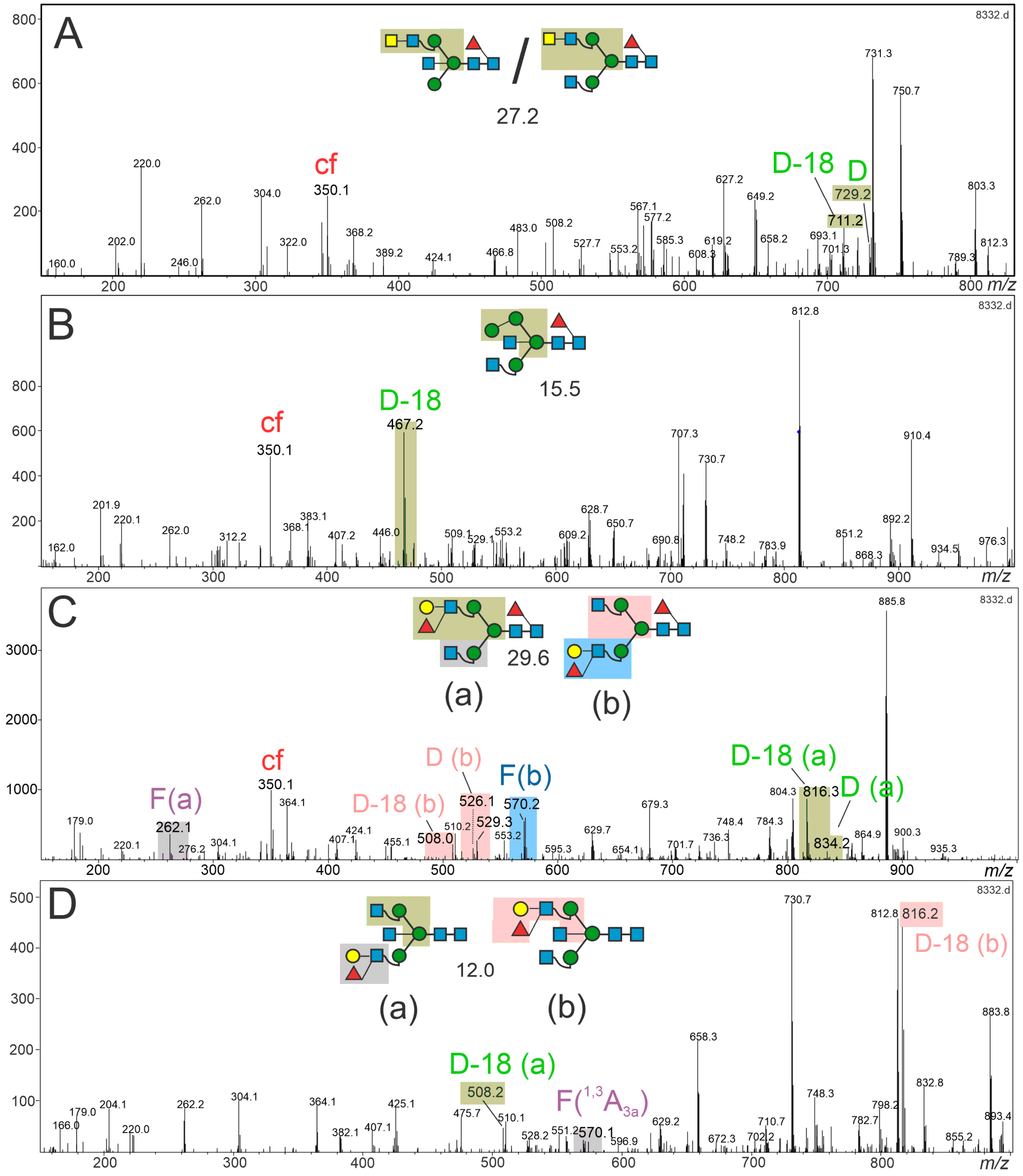


**Figure S11: Negative mode MS/MS spectra of certain N-glycans of the neutral N-glycan pool.** "cf" indicates the 350 *m/z* fragment which is diagnostic for core fucose. The D (and D-18) ions and the F ions denote fragments which are diagnostic for the upper antenna or the lower antenna, respectively [4].

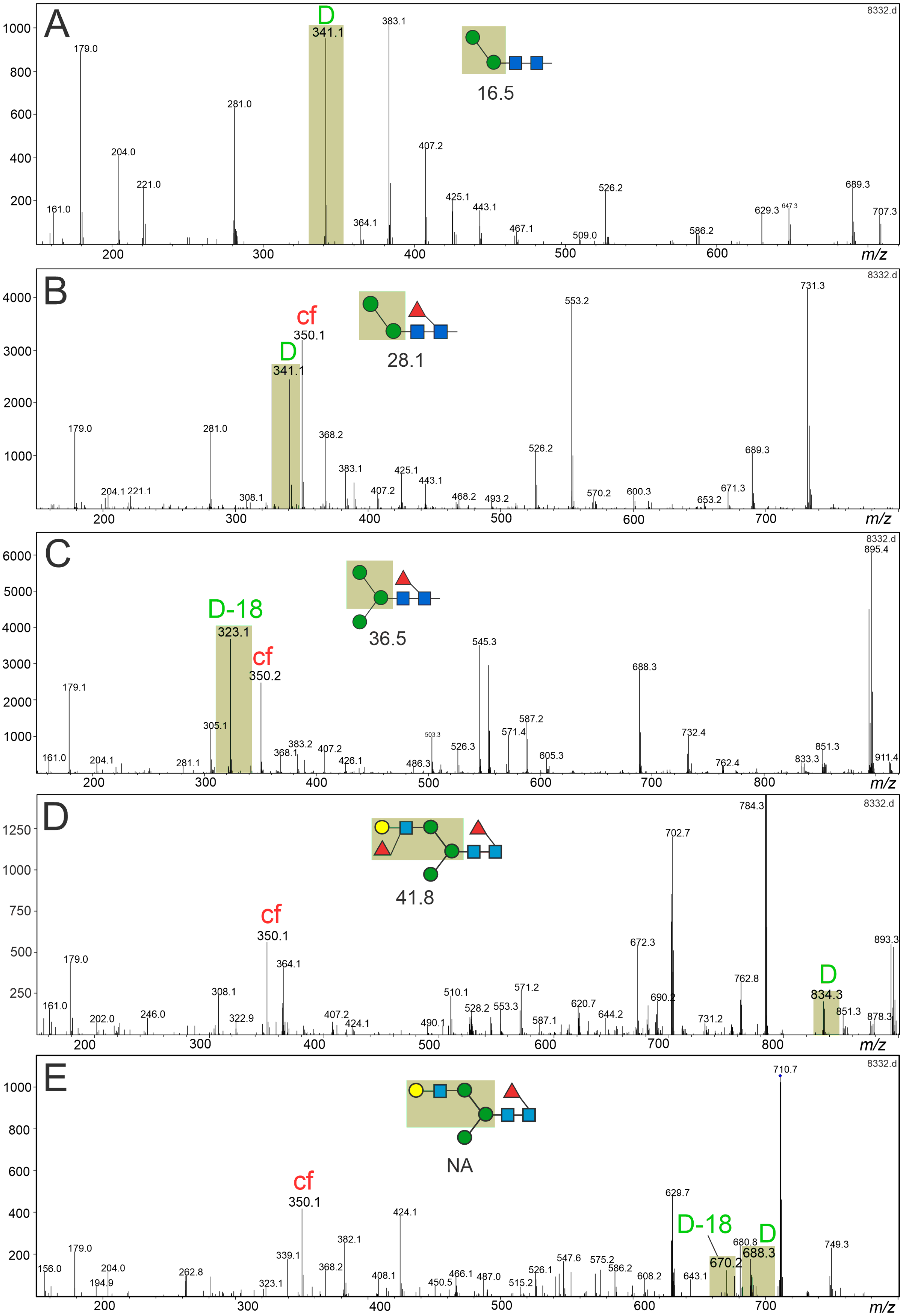

A
D
341.1
383.1
179.0
281.0
204.0
407.2
221.0
161.0
364.1
425.1
443.1
467.1
509.0
526.2
586.2
629.3
689.3
707.3
16.5
8332.d
m/z
B
cf
350.1
D
341.1
553.2
731.3
179.0
281.0
368.2
526.2
689.3
383.1
425.1
443.1
204.1
221.1
308.1
407.2
468.2
493.2
570.2
600.3
653.2
671.3
28.1
8332.d
m/z
C
D-18
323.1
cf
350.2
895.4
545.3
688.3
179.1
305.1
587.2
571.4
732.4
383.2
368.1
407.2
426.1
503.3
526.3
486.3
605.3
851.3
161.0
204.1
281.1
762.4
833.3
911.4
36.5
8332.d
m/z
D
784.3
702.7
41.8
cf
350.1
672.3
893.3
179.0
364.1
762.8
D
834.3
308.1
510.1
571.2
161.0
202.0
246.0
322.9
407.2
424.1
490.1
528.2
553.3
587.1
620.7
644.2
690.2
731.2
851.3
878.3
8332.d
m/z
E
710.7
NA
cf
350.1
629.7
424.1
382.1
D-18
D
680.8
670.2
688.3
749.3
179.0
339.1
156.0
204.0
194.9
262.8
323.1
368.2
408.1
450.5
466.1
487.0
515.2
526.1
547.6
575.2
586.2
608.2
643.1
8332.d
m/z

**Figure S12: Negative mode MS/MS spectra of certain N-glycans of the neutral N-glycan pool.** "cf" indicates the 350 *m/z* fragment which is diagnostic for core fucose. The D (and D-18) ions denote fragments, which are diagnostic for the upper antenna [4].

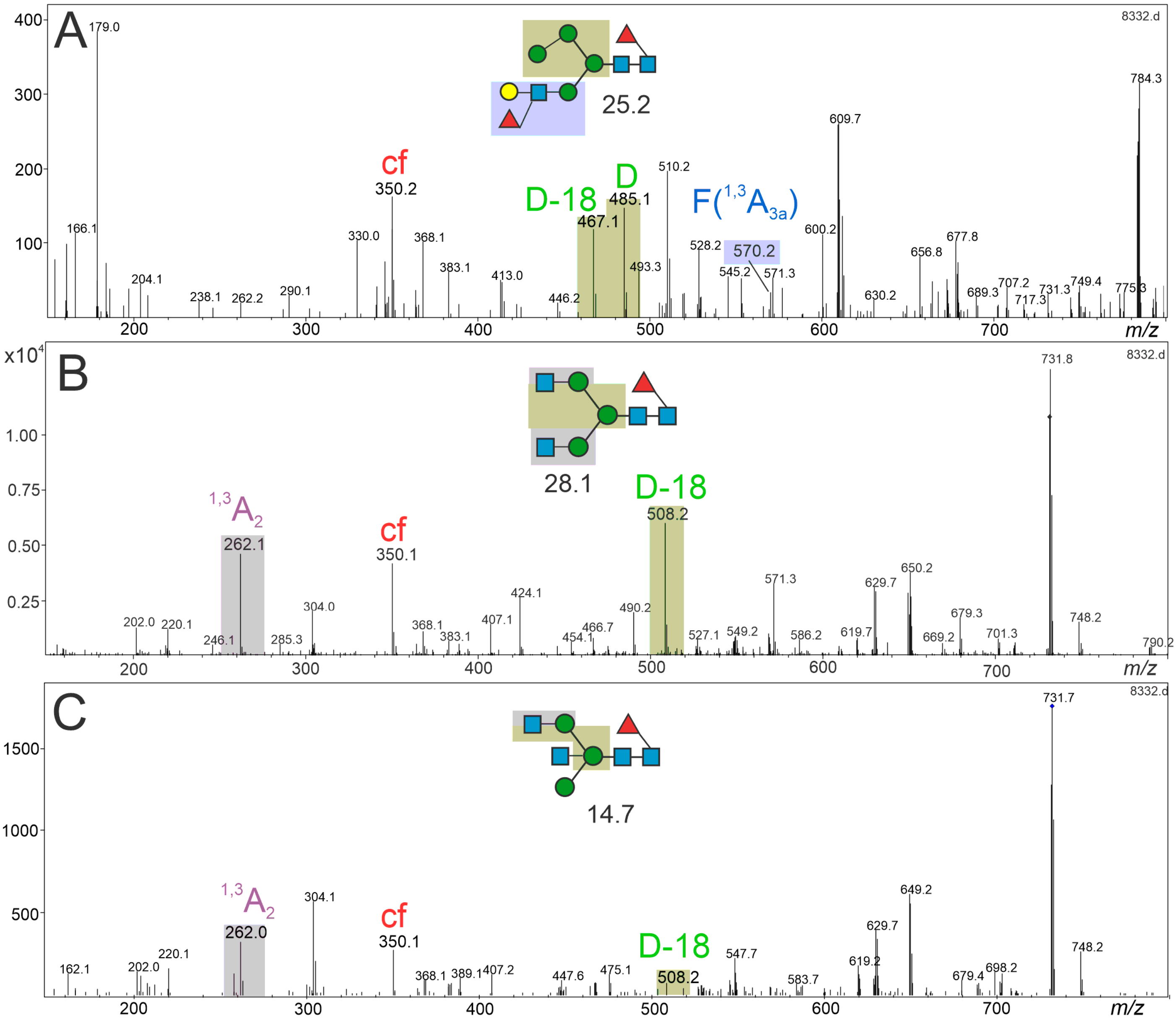


**Figure S13: Negative mode MS/MS spectra of certain N-glycans of the neutral N-glycan pool.** "cf" indicates the 350 m/z fragment which is diagnostic for core fucose. The D (and D-18) ions and the F ions denote fragments which are diagnostic for the upper antenna or lower antenna, respectively [4].

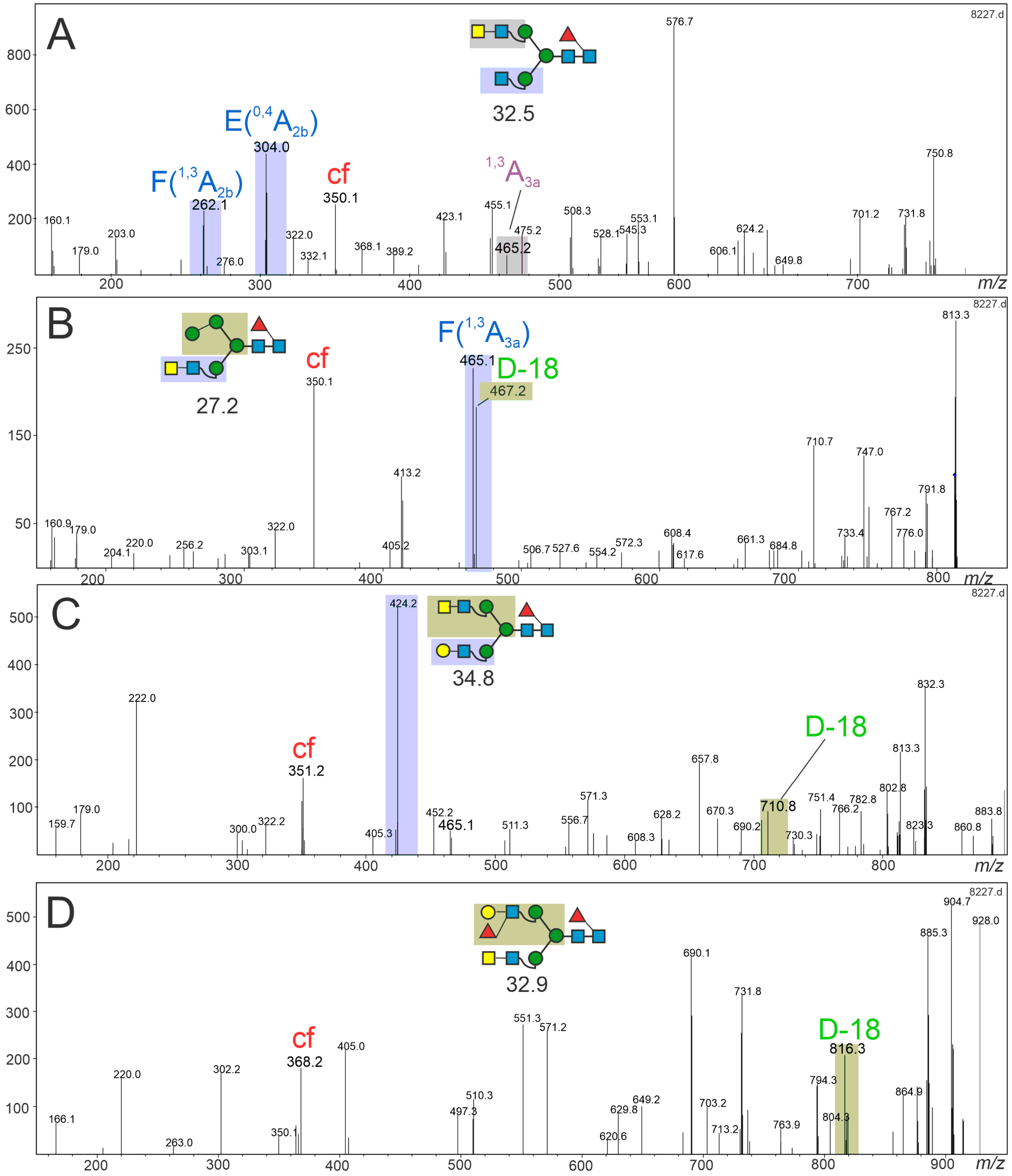


**Figure S14: Negative mode MS/MS spectra of selected N-glycans of the desialylated N-glycan pool**. "cf" indicates the 350 *m/z* fragment which is diagnostic for core fucose. The D (and D-18) ions and the F (and E-ions) ions denote fragments which are diagnostic for the upper antenna or the lower antenna, respectively, as detailed recently [4].

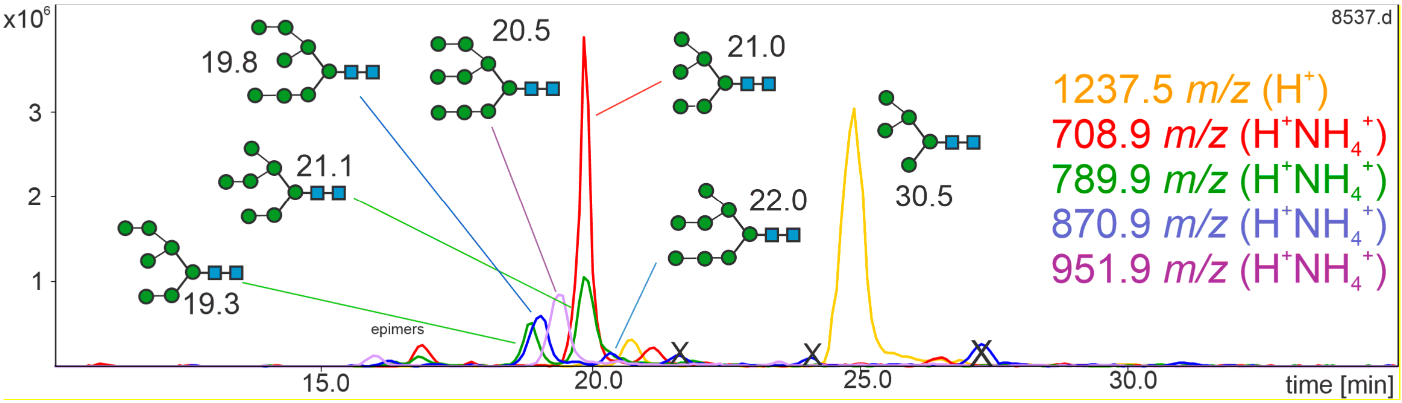


**Figure S15: PGC ESI-MS chromatogram of the oligomannosidic N-glycans from human brain.** Isomer assignment is based on previous work [5].

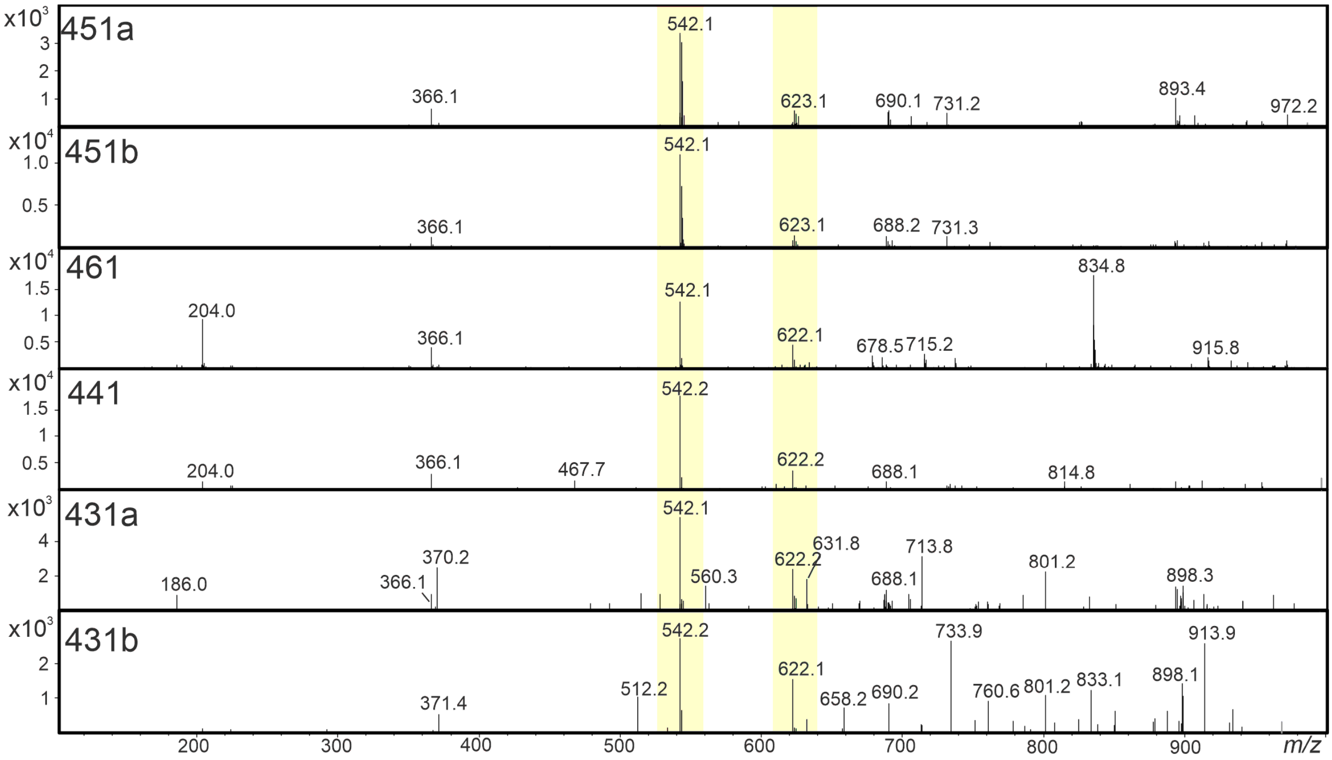


**Figure S16: Positive mode MS/MS spectra of N-glycans from human brain containing the HNK-1 epitope.** Fragments diagnostic for this structur-l feature are marked by color. The diagnostic fragment at 542 *m/z* (GlcA+Gal+GlcNAc) is marked with orange, the diagnostic fragment at 622 *m/z* (SO4+GlcA+Gal+GlcNAc) is marked with green. The associated chromatograms are shown in the main text Figure 5.

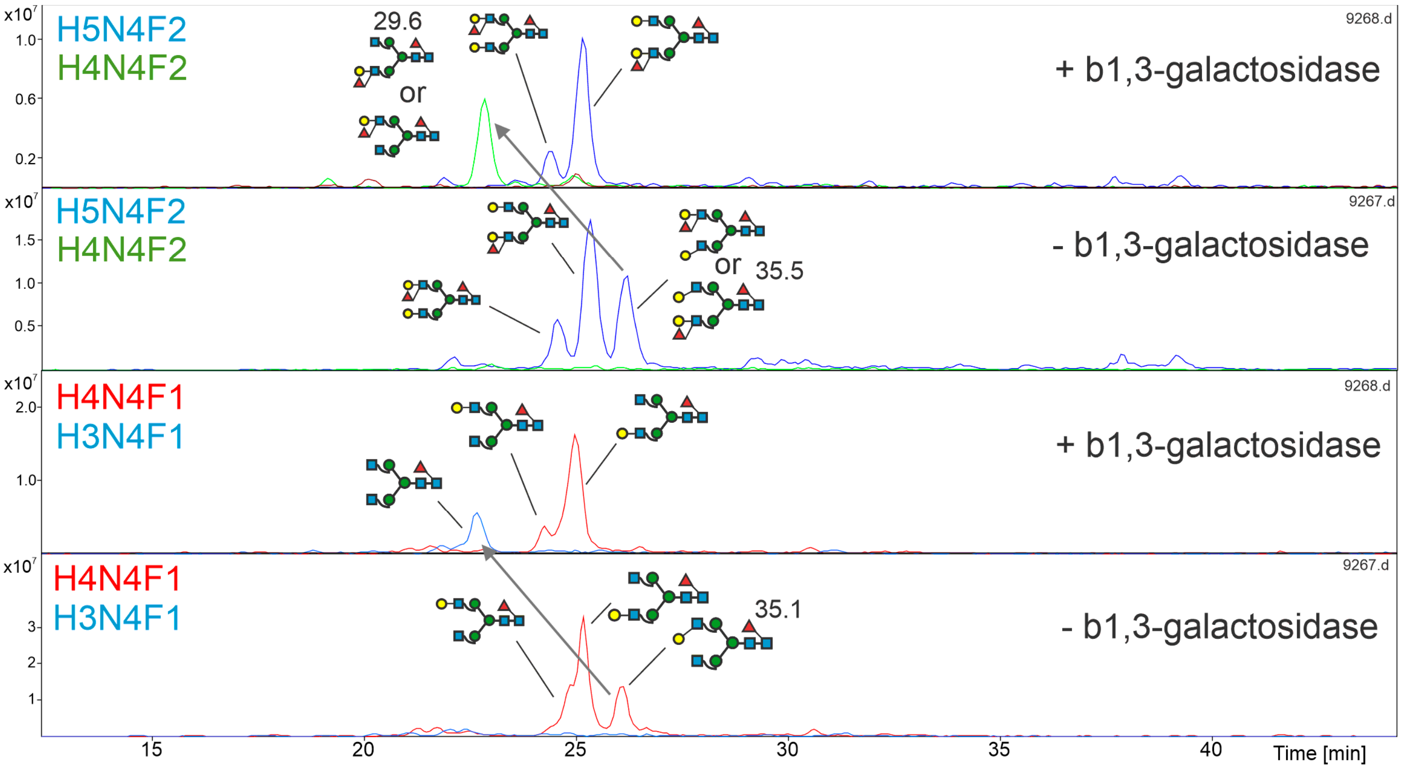


**Figure S17: Relevant PGC-ESI-MS chromatograms of human brain N-glycans before and after β1,3-specific galactosidase digestion.** Two major glycans carrying β1,3-linked galactosidase could be identified (H5N4F2-35.5 and H4N4F1-35.1).

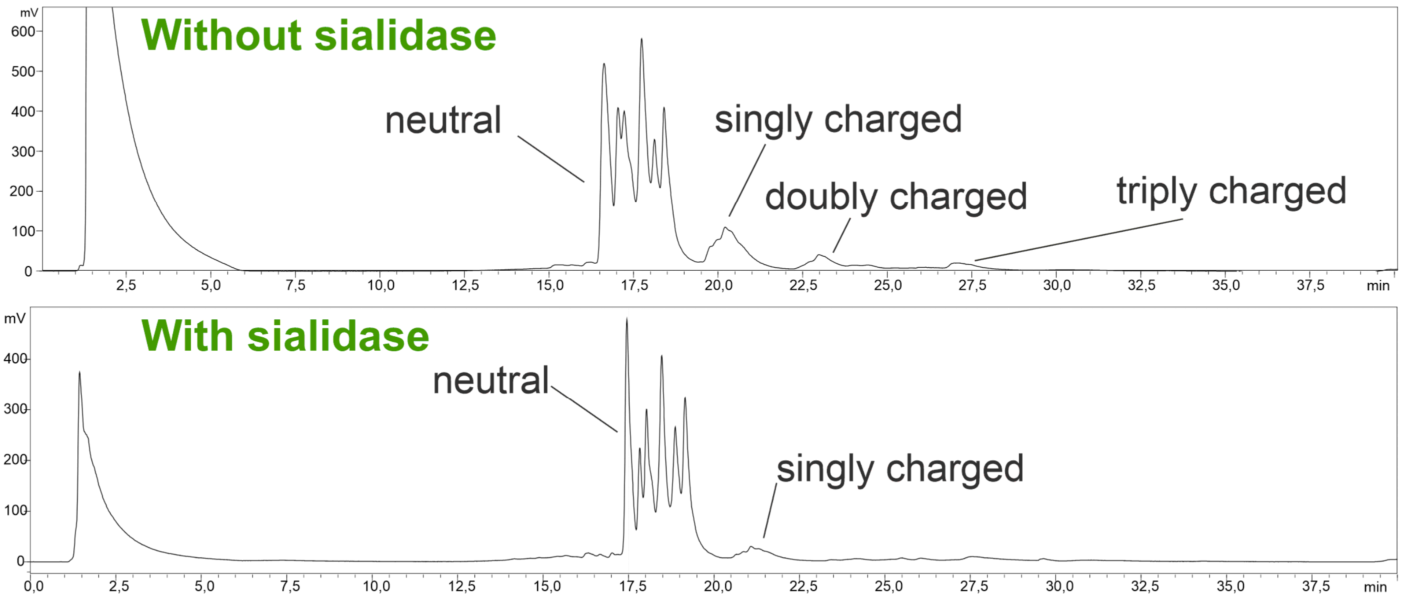


**Figure S18: Mixed-mode HPLC chromatogram of aminobenzamide labelled brain N-glycans before and after sialidase digestion.** The labelled glycans were excited at 330 nm and monitored at 420 nm. The doubly and triply charged fraction completely disappeared after sialidase digestion. The remaining singly charged fraction after sialidase digestion contained charged glycans without sialic acids.

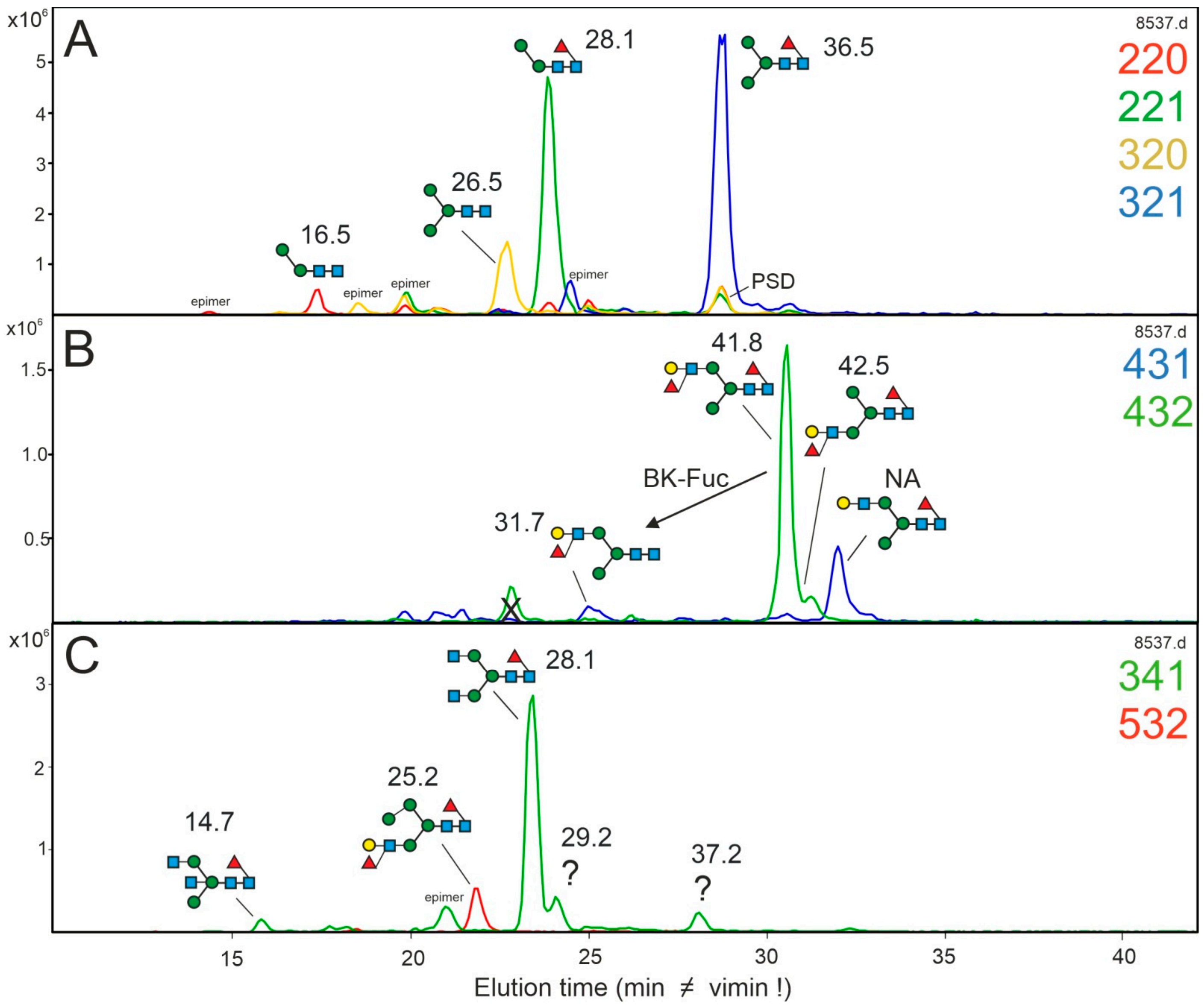


**Figure S19: Elution on PGC of selected smaller brain N-glycans.**

## References:


1. Grass, J.; Pabst, M.; Kolarich, D.; Poltl, G.; Leonard, R.; Brecker, L.; Altmann, F. Discovery and structural characterization of fucosylated oligomannosidic N-glycans in mushrooms. *J Biol Chem* **2011**, *286*, 5977-5984, doi:10.1074/jbc.M110.191304.
2. Guile, G.R.; Rudd, P.M.; Wing, D.R.; Prime, S.B.; Dwek, R.A. A rapid high-resolution high-performance liquid chromatographic method for separating glycan mixtures and analyzing oligosaccharide profiles. *Anal Biochem* **1996**, *240*, 210-226, doi:10.1006/abio.1996.0351.
3. Grunwald-Gruber, C.; Thader, A.; Maresch, D.; Dalik, T.; Altmann, F. Determination of true ratios of different N-glycan structures in electrospray ionization mass spectrometry. *Anal Bioanal Chem* **2017**, *409*, 2519-2530, doi:10.1007/s00216-017-0235-8.
4. Harvey, D.J. Negative Ion Mass Spectrometry for the Analysis of N-Linked Glycans. *Mass Spectrom Rev* **2020**, *39*, 586-679, doi:10.1002/mas.21622.
5. Pabst, M.; Grass, J.; Toegel, S.; Liebminger, E.; Strasser, R.; Altmann, F. Isomeric analysis of oligomannosidic N-glycans and their dolichol-linked precursors. *Glycobiology* **2012**, *22*, 389-399, doi:10.1093/glycob/cwr138.
